# Soft tissue vibration dynamics after an unexpected impact

**DOI:** 10.14814/phy2.13990

**Published:** 2019-01-18

**Authors:** Aaron Martínez, Christopher K.‐Y. Lam, Vinzenz von Tscharner, Benno M. Nigg

**Affiliations:** ^1^ Human Performance Laboratory University of Calgary Calgary Alberta Canada

**Keywords:** Damping, frequency, functional groups, soft‐tissue, vibration

## Abstract

It has been proposed that during walking and running the body has strategies to minimize the soft tissue vibrations. The concept of muscle tuning suggests that muscle activity changes in response to the input signal to modify the frequency and damping of such vibrations. Although it has been demonstrated for continuous vibrations and single impacts, the adaptations dynamics are still unclear. The purpose of this study was to determine (1) if the neuromuscular adaptation to repeated single impacts is immediate, (2) what are the adaptation mechanisms, and (3) if there are functional groups defined by different adaptation strategies. Twenty‐one subjects performed two sets of knee curl on a dynamometer with a custom‐made appliance that supported the foot and heel. The first set was for familiarization with a 90° range of movement and 400°/sec velocity. The second set had 15 repetitions with a 55° range and the same angular velocity. The subjects were not notified of the change; therefore the first impact was unexpected. A pair of electrodes and a three‐dimensional accelerometer were placed on the gastrocnemius medialis. Damping coefficient, natural frequency, and EMG characteristics were measured. All the participants adapted to the vibrations and showed changes in the damping coefficient and or the natural frequency. Apart from the immediate adaptation, a subgroup showed a progressive adaptation after the first immediate change. Three functional groups were identified using support vector machine, correlations with anthropometric values suggest that muscle mass could affect the adaptation strategy used.

## Introduction

Prolonged exposure to vibrations can have detrimental effects on the human body including reductions in motor unit firing rate and muscle contraction (Bongiovanni et al. 1990), decreases in nerve conduction velocity, and reduced peripheral circulation (Dupuis and Jansen 1981; Gilioli et al. 1981; Azmir et al. 2015). During running, vibrations of soft tissue compartments are elicited by the shock wave that is transmitted during impact at landing. Due to the singular nature of the impact and the body's natural tendency to damp vibrations, the duration of the vibrations of the soft tissue packages is relatively short compared to soft tissue vibrations originated by continuous vibration inputs. However, in heel‐toe running, impact shocks often occur between 100 and 200 times per minute (Enomoto et al. 2008) and these input shocks have intensities that are up to five times higher than the corresponding inputs during continuous vibrations such as handling industrial tools or grass cutters (Friesenbichler et al. 2011).

When a soft tissue compartment is exposed to a shock, it vibrates in a damped manner thus reducing the amplitude of the oscillations (Nigg and Wakeling 2001). Nigg and Wakeling (2001) proposed that the body adapts to reduce the vibrations by adjusting muscle activation to the input signal before the expected impact signal occurs. This adaptation was defined as “muscle tuning” (Nigg and Wakeling 2001). Previous research has shown that the body will adapt to different types of input signals, whether it is a continuous vibration that spans a wide range of frequencies (Wakeling et al. 2002), or the impulse of repeated single impacts (Wakeling et al. 2001, 2002; Boyer and Nigg 2007; Enders et al. 2014). To minimize soft tissue vibrations, two principal strategies have been reported. The most reported strategy involves a more rapid damping of the vibration, which is characterized by an increase of the damping coefficient (Wakeling et al. 2002; Friesenbichler et al. 2011). The lesser used strategy is a shift in the frequency at which a system oscillates freely, known as the natural frequency of the soft tissue package (Wakeling and Nigg 2001a).

To our knowledge, only a few studies have assessed how the body adapts to minimize soft tissue vibrations after an unexpected event. Wakeling et al. (2002) had their subjects stand on a vibration platform that could oscillate at different frequencies. Seven pulsed oscillations were applied to the subjects’ feet at six different frequencies, ranging from 10 to 65 Hz. As the order in which the frequencies were applied was random, the first pulse was always unexpected. They observed immediate adaptations already by the second pulse, in the form of: (a) an increase in the vibration damping and power dissipation when the input signal was close to the natural frequency of the soft tissue compartments; and (b) an increase of the total intensity of the electromyography (EMG) in the pre‐activation phase, 50 msec before the applied pulsed oscillation.

In another study, (Boyer and Nigg (2006) assessed the adaptations to vibrations during a dynamic task. Subjects ran on a track that had an unexpected change in the surface hardness. The first step on the unexpected surface did not evoke any changes in EMG intensity, and it was speculated that the leg was not properly tuned to the change in the input signal. There were, however, changes in a multitude of other variables showing some of the consequential effects of the unexpected impact. These included increases in the loading rate, magnitude of the impact force, frequency of the input signal, and peak acceleration of the soft tissue compartments. When examining the second step on the new surface, the researchers did not report significant changes in the EMG pre‐activation intensity, instead they found differences in the leg geometry. They suggested that mechanical reactions such as changes in leg geometry at landing, might provide the necessary adjustments for the transient changes in the running surface properties. These results advocate that a more controlled set up for a dynamic task, where changes in leg geometry are restricted, is needed in order to better understand the adaptations for unexpected events related to muscle tuning.

As evident from the previous studies, adaptations are diverse and unique to the individual (Bongiovanni et al. 1990; Boyer and Nigg 2006; Friesenbichler et al. 2011). However, subject‐dependent responses are not isolated to adaptation strategies for vibrations; they have also been reported with respect to footwear (Schöllhorn et al. 2002; Erhart et al. 2008), running surfaces (Tung et al. 2014) and loading perturbations (James et al. 2014). Individuals whose responses are similar can be grouped as a “functional group” (Nigg 2010), defined as a collection of individuals who react in a similar way to a specific intervention (Hoerzer et al. 2015). Therefore, it is speculated that different groups of individuals will apply different physiological responses when exposed to a repetitive shock wave. However, there is still a need to better understand how the body adapts to unexpected impacts and if there are functional groups with different strategies to minimize the soft tissue vibrations resulting from such episodes.

The first objective of this study was to determine how the body adapts to a series of similar vibrations starting with an unexpected strike, whether it occurs immediately after the first exposure or if the adaptation needs more iterations to occur. The second objective was to assess whether individuals minimize vibrations through changes in the damping coefficient or changes in the natural frequency. The third objective was to define functional groups of individuals that react in a similar way to an expected event, and to determine factors responsible for the grouping. It was hypothesized that:

H1. The adaptation would be immediate.

H2. There would be an adaptation by increasing the damping coefficient and shifting the natural frequency in a subject‐specific manner. However, it is expected that the majority of the adaptation strategies would be via a change in the damping coefficient.

H3. There are functional groups characterized by different adaptation strategies.

## Methods

### Subjects

A total of 21 physically active volunteers participated in this study: 17 males (age 28.5 ± 6.7 years; mass 73.1 ± 6.1 kg; height 177.2 ± 5.3 cm; mean ± SD) and four females (age 24.8 ± 3.3 years; mass 64.3 ± 6.4 kg; height 169.0 ± 4.1 cm). Subjects gave their informed consent to participate in accordance with the University of Calgary's Conjoint Health Research Ethics Board policy on research using human subjects.

### Protocol

**Figure 1:**
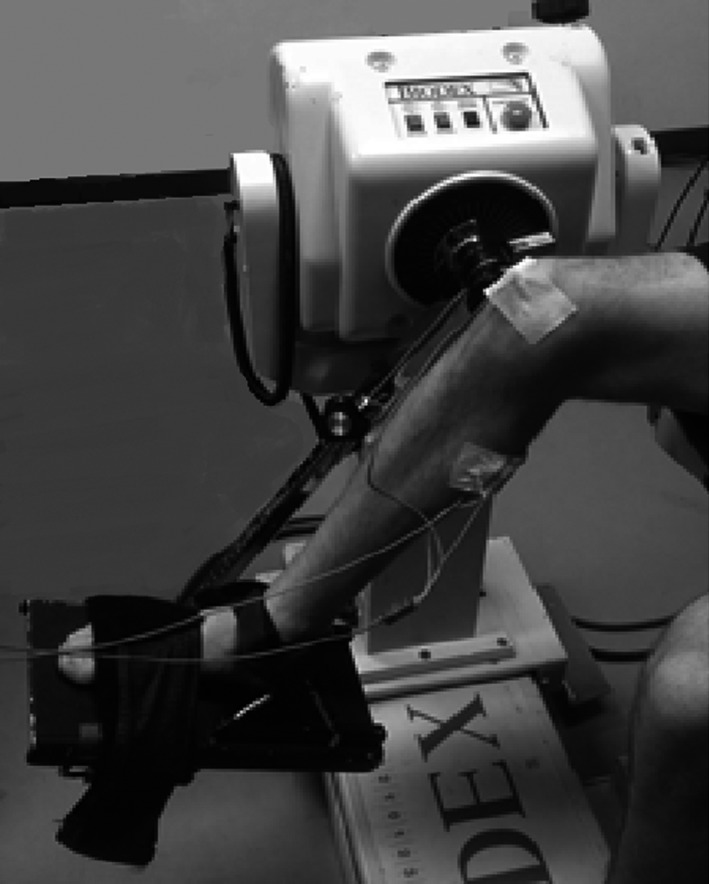
Set up of the right leg with the custom made appliance to support the heel.

The subjects performed two sets of knee flexions on a dynamometer (System 3, Biodex Medical Systems) with a custom made appliance that supported the foot and the heel (Fig. 1). The tests were performed on the right leg. The right knee was aligned with the axis of rotation of the dynamometer and the right thigh was secured to fix the position. No straps were attached to the lower leg allowing free vibrations on the gastrocnemius medialis (GM).

Muscle activation of the GM was assessed by measuring electromyograms (EMG) using bipolar surface electrodes (Ag‐AgCl Norotrode 20, Myotronics Inc., Kent, WA) placed on the muscle belly (according to the Seniam instructions) after cleaning with isopropyl wipes. Each electrode had a diameter of 10 mm, and the interelectrode distance was 22 mm. A ground electrode was placed on the patella. EMG amplifiers with an amplification by a factor of 1000 and a band pass filter from 10 to 500 Hz (Biovision AG D‐61273, Wehrheim, Germany) were used to record the signal at a sampling rate of 2400 Hz. Soft tissue compartment vibrations were measured using a skin‐mounted tri‐axial accelerometer (ADXL 78, Analog Devices, encapsulated in a 20/12/5 mm plastic shell, measuring range ±70 g, frequency response of 0‐400 Hz and a mass < 5 grams) placed 2 cm medial to the electrodes. The accelerometer was attached to the skin using double side tape and stretch adhesive bandage to improve the congruence of motion with the soft tissues. The *y*‐axis of the accelerometer was aligned parallel to the long axis of the shank, the z‐axis normal to the skin surface, and the *x*‐axis normal to the z‐y plane. An additional one‐dimensional accelerometer (ADXL 78, Analog Devices, encapsulated in a 20/12/5 mm plastic shell, measuring range ± 35 g, frequency response of 0–400 Hz and a mass < 5 grams) was placed at the most distal point of the attachment on the dynamometer to determine the phase and timing of the movement. The accelerometer data were recorded with the same DAQ‐card as the EMG and were sampled at 2400 Hz.

The first set of knee flexions was for familiarization and warm up. Subjects began with their leg fully extended and flexed their knee at 400°/sec over a range of 90°. The velocity of the movement was limited by the dynamometer. The subjects were instructed to perform submaximal contractions with a self‐selected force and to apply a constant and consistent amount of force during the concentric phase through all of the repetitions (approximately 15). Recovery to full knee extension was a passive movement controlled by the isokinetic machine. The second set of knee curls consisted of 15 repetitions over a range of 55° at 400°/sec. There was a short break between (<30 sec) the sets in order to start the recording systems. The subjects were not notified of the reduction of the range of motion, therefore the first repetition ended with an unexpected event similar to an impact. After the first unexpected impact, the subjects were informed that the subsequent repetitions would be identical. Due to the similar nature of the sudden break event through all the repetitions, the word impact is used for both, the expected and the unexpected trials. Tri‐axial acceleration and muscle activity were measured during the second set of knee curls. After the testing procedure, anthropometric measurements for shank length and calf girth were conducted. The shank length was identified as the distance between the malleolus lateralis and the lateral knee joint cavity and calf girth was measured around the thickest part of the calf, approximately 1/3 from the proximal knee joint.

### Vibration analysis

Previous studies have shown no significant effect of gender on muscle tuning (Wakeling et al. 2002); therefore all subjects were pooled together for the analysis. The soft tissue mass was considered as one rigid mass for the analysis. Accelerations in the three axes of the accelerometer were analyzed using a custom made code in MATLAB (Version 8.6.0, The MathWorks Inc., Natick, MA).

**Figure 2:**
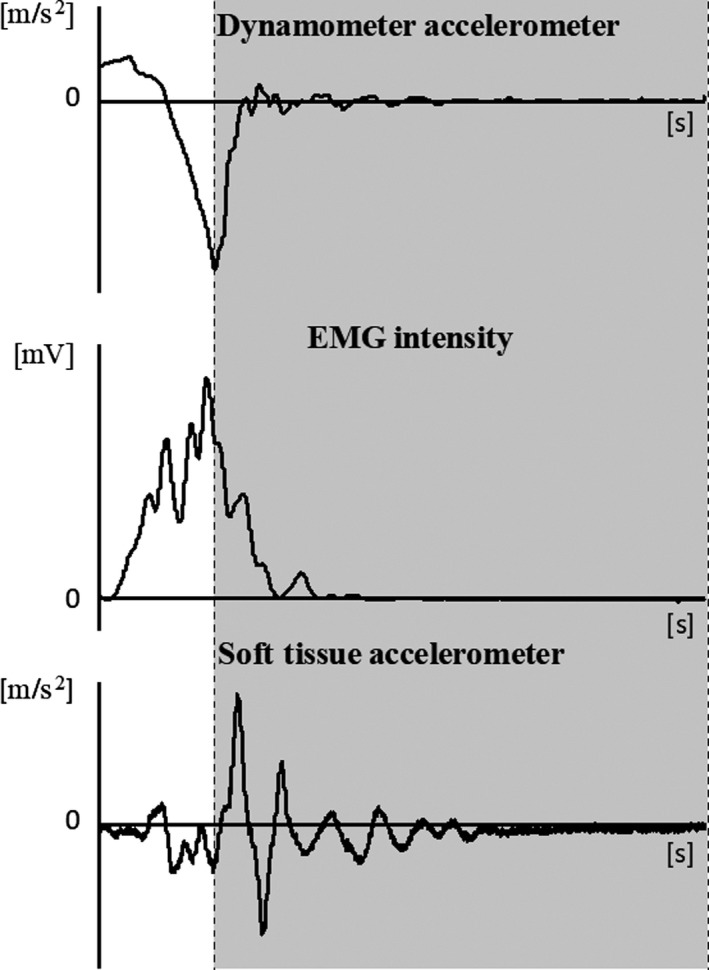
Acceleration data from the one dimension accelerometer placed at the end of the appliance, EMG intensity for the GM and acceleration data from the three dimensions accelerometer placed over the GM, from top to bottom, respectively. The shaded part represents the time window of one‐second, starting at the beginning of the breaking phase of the dynamometer arm, used for the analysis.

The signal was bandpass filtered using a wavelet filter (von Tscharner 2000) from 2 to 50 Hz (Table 1). The range of frequencies was chosen according to the reported range of natural frequencies for the lower limb soft tissue compartments (Wakeling and Nigg 2001a,b). After filtering, the signal was cut from the onset of the vibrations to 0.85 sec thereafter and was used for the analysis (Fig. 2). The onset of the vibration was determined as the moment of the impact. It was detected as the peak in the acceleration signal from the accelerometer placed on the arm of the dynamometer. The 0.85 sec were selected in order to ensure that the vibrations were minimized and to ease further calculations. The cut signal was duplicated, reversed, and mirrored to the front end in order to obtain a continuous wavelet, one that lacked a sharp edge, needed for further analysis. After the wavelet analysis, the previously added mirrored data were removed from the front end of the continuous signal.

**Table 1:** Characteristics of the wavelets used for the vibration analysis and myoelectric analysis. Scaling factor of 1. The first wavelet was excluded from the analysis

	Vibration analysis		Myoelectric activity analysis	
Wavelet index	Center frequency [Hz]	Time resolution [ms]	Center frequency [Hz]	Time resolution [ms]
1	2.07	269.2	6.9	79.2
2	5.79	176.6	19.3	51.7
3	11.31	128.3	37.7	37.5
4	18.63	100.8	62.1	29.2
5	27.71	83.3	92.4	24.2
6	38.54	70.8	128.5	20.0
7	51.12	60.8	170.4	17.5
8	65.42	54.2	218.1	15.8
9	81.45	48.3	271.5	13.6
10	99.19	40.0	330.6	12.1
11			395.4	10.8
12			465.9	9.2
13			542.1	7.1

The frequency content of the vibrations that were elicited by each event was assessed using a Fast Fourier Transformation (FFT). The natural frequency of the soft tissue compartment was estimated by the highest peak value within the power spectrum (0.6 Hz resolution). To measure the decay of the power, a filter bank of 10 non‐linearly scaled wavelets was used for the wavelet transform (Enders et al. 2012). Center frequencies and time resolution of those wavelets are shown in Table 1. The wavelet transformation consisted of a convolution of the signal with the real and imaginary part of each wavelet. The power was obtained by adding the squared values of the real and imaginary part of the wavelet transformed signal. The overall power was the sum of the power extracted from all the wavelets. The decay of the overall power over time, which is independent of the frequencies, was best described by an exponential function (equation 1).

(1) $$p\left(t\right)=P_{0}e^{-dt}$$

P_0_ [ms^−2^] was the power of the signal just after the impact and the damping coefficient, d [s^−1^], was a measure of how rapidly the power of the vibration decays over time. The numeric value of the damping coefficient was obtained by fitting an exponential function to the measured decay of the power using a least squares method (Wakeling and Nigg 2001a).

The analysis resulted in two variables for each of the 15 events, the natural frequency extracted from the power spectrum and the damping coefficient extracted using the wavelet transform. The mean and standard deviation characterized the distribution of the variables not including the values for the first unexpected events. To resolve whether the adaptations in the damping coefficient and natural frequency occurred immediately after the first unexpected event, we tested whether the probability that the variables of the first repetition belonged to the distribution of the later ones. If the variables were outside of the 95% range determined by the mean ± 2 standard deviations the adaptation was deemed immediate.

### EMG analysis

The myoelectric signals were resolved into their intensity in time‐frequency space by use of a wavelet analysis (von Tscharner 2000). A set of 13 wavelets was used (Table 1) and the overall intensity was calculated across wavelets 2‐12, which is equivalent to band‐pass filtering the signal between 19.3 and 465.9 Hz (von Tscharner 2000).

The time window for the EMG analysis started at the beginning of the breaking phase of the movement (Fig. 2), characterized by a peak in the acceleration data of the accelerometer placed on the arm of the dynamometer, and was one‐second long. The muscle was considered to be active when the EMG intensity was higher than 5% of the peak value for each trial.

### Functional groups

In order to determine if there were functional groups with different adaptation strategies, a label was assigned to the measured variable. The label was 1 when the outcome was a significant change (*P *<* *0.05) in the expected direction (an increase in damping coefficient, a decrease in EMG duration and/or a decrease in EMG intensity), it was 0 when there was no significant change, and it was ‐1 when there was a significant change in the opposite direction. An orthogonal vector space of dimension *N* = 18 was constructed whereby each variable (damping coefficient and natural frequency in x, y, and z axes, EMG duration, intensity, and mean frequency, all of them immediate and non‐immediate) is represented by one axis. The values on the axes were the labels mentioned above. In this vector space a k‐means clustering was performed based on the Euclidian distances to separate the groups (Bishop 2006). To decide how many clusters would be optimal, the procedure was done for four cases, assuming two, three, four, and five clusters. The sum of the squared distance between each member of the cluster and its centroid was used to assess the quality of the arrangements. The arrangement with the lower value out of 50 random initializations for each k‐means cluster analysis was selected. A silhouette analysis was used to determine the optimal number of clusters (therefore groups) that could be separated (Rousseeuw 1987). The silhouette analysis yielded a value “s” between +1 and ‐1 for each subject in a cluster indicating whether the subject belonged to this cluster. A positive “s” indicated that the subject was a member of the group, an “s” around 0 reflected an undefined classification whereas a strong negative value indicated a misclassification. The optimal number of groups was estimated by selecting the case with the number of clusters that minimized the number of misclassifications and maximized the median of the “s” values of the clusters. These groups are called adaptation groups because they adapt to the impact in a similar way, and therefore, they represent functional groups.

Together, the labels of the variables were represented in an N (18) dimensional vector space. However, some of the 18 variables (damping coefficient and natural frequency in x, y, and z axes, EMG duration, intensity, and mean frequency, all of them immediate and non‐immediate) may respond in a correlated way to an intervention. The means of the variables that were obtained for the 21 subjects were subtracted from the measured ones and normalized by dividing them by their standard deviation. A principal component analysis (PCA) was applied to a matrix representing the normalized variables in an 18‐dimensional vector space (Trudeau et al. 2015). The PCA yielded the PC‐vectors, which indicated variables that contributed in a correlated way to the individual measurements, and the weights that indicated how much each PC‐vector contributed to the measurements obtained from one subject. For the analysis, only the weights of the first two PC‐vectors were considered because they explained most of the variability of the weights and were enough for grouping the subjects in a reduced, two‐ dimensional vector space.

### Statistical analysis

Regression slope *t*‐tests were employed to evaluate if there was a continuous change in the damping coefficient and the natural frequency values. Thus, indicating a progressive adaptation (see Results section).

In order to assess the significance of the separated groups, classification rates in combination with a one‐sided binomial inverse cumulative distribution test (Enders et al. 2014) were used. The classification rates were determined by a leave‐one‐out cross‐validation that was applied using a Support Vector Machine (SVM) (Maurer et al. 2012; Hoerzer et al. 2015).

Finally, to determine whether there were differences in the anthropometric measures between the groups, the shank length, calf girth and a ratio between shank length and calf girth were compared between the groups using a one‐way analysis of variance (ANOVA). A significance level of 0.05 was chosen.

## Results

All of the participants adapted to the vibrations and showed changes in the damping coefficient and or the natural frequency.

When examining whether the adaptations were immediate, some subjects showed a different trend. They did not adapt immediately but progressively over subsequent impacts. Moreover, some of the participants showed a combination of both behaviors, they had an immediate adaptation but they continued to adapt over repeated strikes. Consequently, in the following paragraphs it will be specified if the adaptations found in the damping coefficient and in the natural frequency where immediate, progressive or a combination of both.

### Damping coefficient

**Figure 3:**
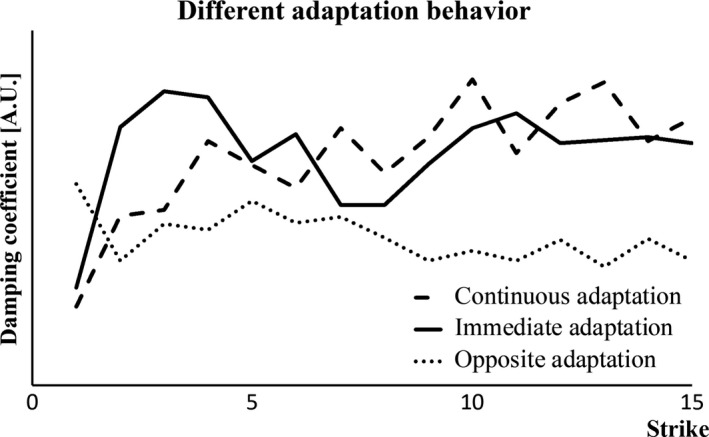
Damping coefficient values over 15 strikes. The solid line is an example of a subject with an immediate adaptation, with a big change between the first and the second strike and no further adaptation. The dashed line is a progressive adaptation, it has a tendency to increase the value over subsequent strikes. The dotted line shows the opposite to the expected behavior, the value of the damping coefficient decreases over the repeated strikes.

Significant changes in the damping coefficient in the *y*‐axis (parallel to long axis of the leg) were found in 18 of the 21 participants. Of these 18 participants, 17 adapted immediately to the vibration while one participant showed a progressive adaptation. Although most participants had a significant adaptation already by the second impact, there was a subgroup consisting on 11 of those 17 participants that continued to adapt over subsequent strikes, thus also showing a progressive effect. Not all of the participants had an increased damping behavior with repeated exposure, in fact five subjects had a decrease in the damping coefficient with repeated exposure (Fig. 3).

When examining how adaptations occurred in each axis, 13 participants demonstrated changes in the *x*‐axis (mediolateral axis), eight were immediate and five were progressive. Of these 13 participants, nine showed an increase in the damping coefficient while four showed the opposite behavior by decreasing the damping coefficient. For the axis normal to the skin (z‐axis), 17 subjects showed an adaptation, where 10 were immediate and seven were progressive. Of these 17 subjects, nine subjects increased and eight subjects decreased the damping coefficient.

### Natural frequency

A shift in the natural frequency was found in 15 of the 21 subjects in the *y*‐axis. Of these 15 participants, 10 showed an immediate change and the rest had a progressive shift in the natural frequency. The same change was seen in 13 participants for the *x*‐axis, with six that had immediate and seven that had progressive changes. Finally, for the z‐axis, 15 participants showed adaptations, 13 had immediate changes, and two had progressive changes.

### Myoelectric activity

The duration of the muscle activation was reduced in 18 subjects, corresponding to a reduction in the total intensity. Total intensity was calculated after the impact, consequently the active phase involving the voluntary movement and the preactivation phase were not included. Fifteen participants already had a significant reduction in the duration by the second impact (immediate change), while three had a progressive adaptation. The reduction in duration was reflected in the EMG intensity analysis where 19 participants showed a significant decrease in EMG total intensity (post movement). Seventeen participants changed immediately while two had a progressive adaptation.

### Functional groups

The “s” index used to determine the selected number of groups showed negative values for the two groups clustering, therefore it was not used for further classification. The 3‐groups classification was selected over the 4‐groups classification as it presented a higher median value (3: 0.2417 vs. 4: 0.2415).

All subjects but one were assigned correctly to their group (defined by the k‐mean clustering analysis) according to the leave‐one‐out cross validation approach using a SVM (von Tscharner 2000). There was a significant probability (*P* < 0.01) of correctly classifying 96% of individuals with the SVM.

**Figure 4:**
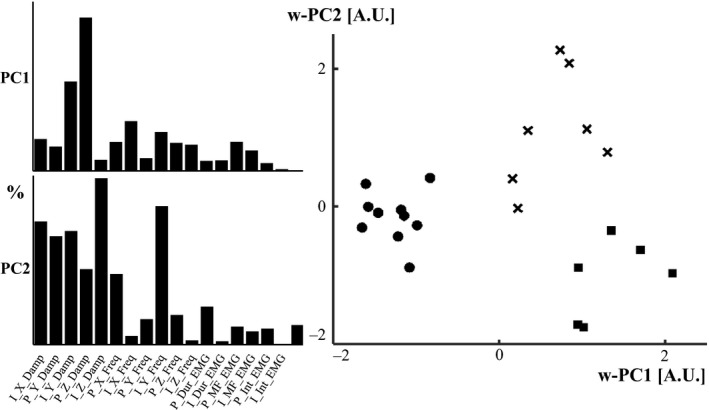
On the left side there is the representation of the contribution of each variable to the unitary value of the two principal components. PC“x” = Principal component “x”; P  =  Progressive adaptation; I  =  Immediate adaptation; X  =  *x*‐axis; Y  =  *y*‐axis; Z  =  z‐axis; Damp = Damping coefficient; Freq = Frequency shift; Dur = Duration; MF = Mean frequency; Int = Intensity. For instance, P_X_Damp means: progressive adaptation on the X axis of the damping coefficient. On the right side there is the distribution of the subjects of the three different groups determined by the principal components 1 and 2. w  =  Weight. The circles represent the first group, characterized by an increase in the *y*‐axis of the damping coefficient. The squares represent the second group, characterized by a shift in the z‐axis of the natural frequency. The crosses represent the third group, characterized by a decrease in the *y*‐axis of the damping coefficient.

The results of the PCA showed that the damping coefficient contained the variables responsible for the majority of weight defining the two principal components, primarily in the *y*‐axis, followed for the z‐axis (Fig. 4 left). It was found that the first (PC1) and second (PC2) principal component's weights were able to separate the three groups previously defined (Fig. 4 right).

The largest of the three groups was comprised of 10 subjects, all of which showed an immediate adaptation in the *y*‐axis of the damping coefficient. Furthermore, seven of the 10 subjects also had a progressive adaptation to the same variable, meaning they not only had a significant immediate change, but that they were able to keep adapting afterwards. Changes in the intensity and duration of the EMG were also found in all 10 subjects of this group, eight of which showed immediate reactions while two of them had a progressive reaction. This adaptation in muscle activity clearly reflected the behavior of the adaptations in the *y*‐axis of the damping coefficient of this group. There were no other similarities in the rest of the variables assessed.

The second group, formed by six individuals, was primarily characterized by changes in the z‐axis for both damping coefficient and natural frequency. For the damping coefficient, all of the participants showed an adaptation, five of them had immediate adaptations (two of which also showed further adaptation beyond the second strike) and the last participant had a progressive adaptation. For the natural frequency, all participants had an immediate change, and moreover, four of them kept adapting. In this second group the EMG signal reflects the behavior of the z‐axis of the natural frequency. It showed either immediate or immediate and progressive adaptations simultaneously in both variables. Five of the six individuals in the group had a non‐immediate adaptation in the *x*‐axis of the natural frequency. Interestingly, the ratio between shank length and girth was statistically higher for this group compared to the other two (*P* < 0.05).

The last group was made up of five participants. This group was predominantly characterized by a decrease of the damping coefficient in the y and x axes, as well as no adaptation in many of the other variables. No similarities were seen for the natural frequency variables assessed. Three of the five subjects had adaptations in the intensity and duration of the EMG signal.

## Discussion

This study assessed the adaptations of the soft tissue vibrations over subsequent similar shocks after a first unexpected shock. We wanted to better understand which mechanisms contribute to the fast damping of the soft tissue vibrations, and how is the process over time.

### Immediate versus progressive adaptation

Our results corroborate the findings by Wakeling et al. (2002) where participants show immediate adaptations to repeated strikes that generate vibrations through the soft tissue. The number of subjects presenting an immediate adaptation predominated through all variables. Surprisingly, for the participants that showed an immediate adaptation, a subgroup (50%) showed a progressive adaptation even after the first immediate change was identified. The progressive adjustment following the first immediate change showed an adaptation that has never before been reported. This exemplifies the body's ability to rapidly protect itself from potentially damaging inputs and furthermore, the ability of progressively tune itself with prolonged stimulus. The adaptations were assessed on the GM, a muscle directly involved on locomotion. In consequence, this particular muscle has been exposed to vibrations uncountable times and has developed an efficient mechanism to minimize them. Although the direction of the impact was not the same than during locomotion, the transmission of the vibrations is similar, it starts in the ankle and travels through the leg to the calf. That could be the reason why most of the subjects were able to adapt as quick as after one single shock. We believe that the body has some threshold where vibrations are considered harmless; therefore, the subjects that after the first impact accomplished to minimize the vibrations under that point, did not need further adaptation. On the other hand, the participants that were still over the threshold needed to keep tuning the muscles in order to better protect the tissue. Some subjects seemed to reach a plateau after some trial, reinforcing our idea of a threshold; however, further research is needed in order to confirm that behavior.

### Method of adaptation: damping coefficient or natural frequency

Previous research has shown two different mechanisms used to minimize vibrations. Increases in the damping coefficient (Wakeling et al. 2002; Friesenbichler et al. 2011) has been vastly more reported than shifts in the natural frequency (Wakeling and Nigg 2001a). Therefore, we hypothesized that adaptations would be subject specific and found mostly on damping coefficient, but some subjects would adapt on the natural frequency. The results ratified the hypothesis, as adaptations were identified in both natural frequency and damping coefficient with a predominance of the later. However, the natural frequency was used by 71% of the participants. (see 4.3). The type of task selected for the test might have influenced which adaptation mechanism was used. Considering that the movement is submaximal, predetermined and guided, and the leg position fixed, it seems feasible the use of a change in the initial tension of the muscle as adaptation mechanism. The change in the initial tension would alter the natural frequency of the soft tissue package without necessarily modifying the movement. As aforementioned, the GM is a muscle already adapted to locomotion. Walking and running need more control from the muscles than the used knee flexion task, probably affecting the chosen mechanism to minimize vibrations. On the other hand, it seems reasonable to argue that if some subjects tend to use an increase on the damping coefficient as a protective mechanism during locomotion, they probably keep using it when performing a new task. To better understand the predominance of the different adaptation mechanisms, it is necessary to determine which variables are more representative of the adaptation and if the different strategies to reduce the vibrations can be classified.

### Functional groups

The largest group was characterized by adaptations of the damping coefficient in the *y*‐axis (longitudinal axis). As these individuals did not show a consistent change in the natural frequency, they were defined as “dampers”. The PCA proved this axis to have the most weight in the first principal component, thus determining the variable with the higher variance and representing the principal factor defining the groups. It was expected that this would be the axis to predominantly show adaptations as the transmission of the vibrations evoked by locomotion, from heel to the upper sections of the body, occurs along this axis. This prediction was confirmed by the percentage of significant changes being the highest in this direction and by defining the variable with the most variance determined by the PCA.

A second group given by the clustering analysis was predominately characterized by changes in the natural frequency. The changes in the natural frequency were inherent in multiple axes, with the z‐axis being the predominant one, and found in all subjects within the group. We defined them as “frequency shifters”. Although those changes were the governing features, some individuals in this group also showed changes in the damping coefficient (z‐axis).

Nonetheless, some subjects classified in one of the previous groups presented the defining characteristics of both groups. This is an indicator that adaptations are not limited to either changes in the damping coefficient or in the natural frequency as previously reported (Boyer and Nigg 2007), but that some subjects adapt with a combination of both mechanisms. The fact that the muscle tone at the onset of the vibrations might be altered to produce a shift in the natural frequency does not necessary limit the appearance of other protective strategies (e.g., an increase in the damping coefficient) that might occur concurrently with the vibrations.

**Figure 5:**
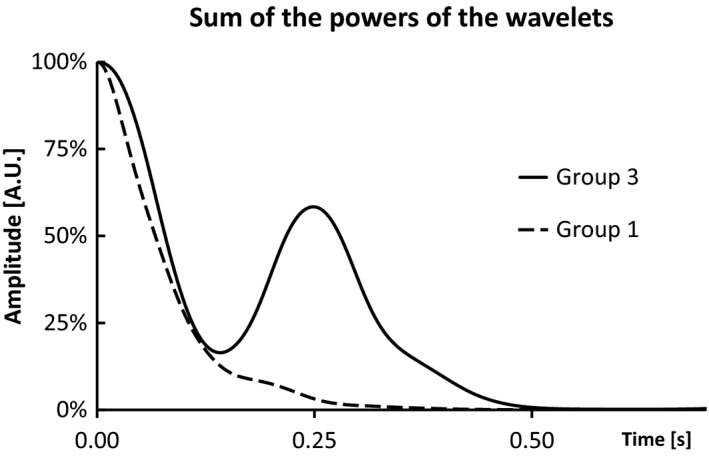
Sum of the powers of the wavelets for a subject from the 1st group (damper) and a subject from the 3rd group. The second peak is a characteristic of the subjects affected by the beating.

Furthermore, the third group identified by the cluster analysis was also characterized by the *y*‐axis of the damping coefficient. Those individuals showed an opposite behavior to what has been reported before, they presented a decrease in the value of the damping coefficient. Although the damping coefficient decreased, the amplitude of the first oscillation for all strikes were similar and the vibrations minimized faster with repeated exposure. This phenomenon could be caused by the anisotropy of the human soft tissue packages. The individuals in this group also showed a consistent shift in the natural frequency of the z‐axis. This modulation of the muscle activity possibly caused a change in the anisotropy characteristics of some subjects leading to a more complex behavior known as beating. When there are different oscillations affecting the same system and their frequencies are close together the interaction between those various frequencies leads to an overlap, potentially canceling the waves and minimizing the vibrations. When analyzing the raw acceleration signal, the oscillations do not reproduce the characteristic damped behavior, and this is translated to a characteristic shape in the sum of the powers of the wavelets. The subjects in this group presented a second minor peak in the sum of the wavelets’ power (Fig. 5) causing the decrease in the damping coefficient although the vibrations were minimized faster. This effect might be caused by the interaction of the multimodal vibration signal. Depending on the phase between waves, they might be canceled or not enhanced. Consequently, the dampening effect of beating seems not to be constant.

### Anthropometric differences between groups

It has been previously suggested that the muscle mass might define the type of adaptation. Boyer and Nigg (2007) theorized that muscle tuning might occur in the natural frequency and not in the damping coefficient for smaller muscles within the same subject. Following this line, we compared various anthropometric measurements between the groups. A significantly higher ratio between shank length and calf girth, which could be an indicator of an increased muscle mass, was found in the second group, “the frequency shifters”, compared with the other two groups. It suggests that larger muscles might use natural frequency shifts as a preferred adaptation mechanism to minimize vibrations. Those differences represent a first step to better understand what determines the strategies utilized to minimize vibrations in the soft tissue packages.

### Novelty of study design

Our results differ from those reported by Boyer and Nigg (2007) and the reason behind it could be due to the difference in both tasks. In the previous study, subjects struck a force plate while lying motionless on a pendulum bed, whereas in this study, our subjects performed a dynamic task contracting the muscles voluntarily. This difference is based on the need for a dynamic and controlled set up where the subjects voluntarily activate their muscles, which is more similar to what happens while walking or running. Attaching the subjects to the dynamometer and limiting the range of motion and the speeds ensures an environment where the input is consistent, enables the body to adapt over several repetitions. As mentioned in Boyer and Nigg (2006) in the hierarchy of motor control tasks, the muscle tuning response is expected to be lower on the priority list of tasks to be accomplished during the landing phase of gait. With the controlled set up it was ensured that the leg geometry was constant, and suggested that muscle tuning could occupy a higher position in the propriety of tasks. The methodology had some limitations due to the nature of the movement and the test. The movement was very fast (400°/sec), limiting the option to give feedback to the subjects in order to control the consistency of the movement. To cope with that problem and with the impossibility of repeating the measurements, every subject performed enough repetitions during the familiarization in order to feel comfortable and consistent though all the repetitions. Since subjects performed an active dynamic task, it was difficult to differentiate what percentage of the muscle activity was used for pushing the dynamometer and what was meant for minimizing vibrations. However, with an appropriate preactivation (Wakeling et al. 2002), the muscular activity needed after the impact might be reduced, and thus, a lower EMG intensity or duration would be an indicator of an adaptation prior to the actual impact. As a consequence, were able to understand the mechanisms and timings for the different strategies adopted to minimize vibrations on the soft tissue packages.

## Conclusion

To date, the mechanisms to minimize vibrations in the soft tissue packages were traditionally described as an increase in the damping coefficient or a shift in the natural frequency values. The results of this study showed that some subjects use both mechanisms at the same time and suggest that other mechanisms, as the beating effect, may also affect.

The capacity of some subjects to continue adapting progressively after an immediate change is another finding never before reported.

The strategies utilized by the subjects characterizing various functional groups and the differences in anthropometrics between those groups represent a promising approach to find the mechanisms behind which strategy is used by every individual.

## Conflict of Interest

The authors declare no conflicts of interest, financial or otherwise.
